# The Effect of Surface Confined Gold Nanoparticles in Blocking the Extraction of Nitrate by PVC-Based Polymer Inclusion Membranes Containing Aliquat 336 as the Carrier

**DOI:** 10.3390/membranes8010006

**Published:** 2018-01-25

**Authors:** Ya Ya N. Bonggotgetsakul, Robert W. Cattrall, Spas D. Kolev

**Affiliations:** School of Chemistry, The University of Melbourne, Victoria 3010, Australia; yayabong88@gmail.com (Y.Y.N.B.); r.cattrall@unimelb.edu.au (R.W.C.)

**Keywords:** polymer inclusion membrane (PIM), gold nanoparticles (AuNPs), surface morphology, Aliquat 336, nitrate extraction

## Abstract

Clusters of gold nanoparticles (AuNPs) formed on the surface of PVC-based polymer inclusion membranes (PIMs) with a liquid phase containing Aliquat 336 as the carrier and in some cases 1-dodecanol or 2-nitrophenol octyl ether as plasticizers were found to inhibit the extraction of nitrate by the PIMs. This observation was based on gradually increasing the mass of AuNPs on the membrane surface and testing the ability of the membrane to extract nitrate after each increase. In this way, it was possible to determine the so-called “critical AuNP masses” at which the studied membranes ceased to extract nitrate. On the basis of these results, it can be hypothesized that the surfaces of these PIMs are not homogeneous with respect to the distribution of their membrane liquid phases, which are present only at certain sites. Extraction takes place only at these sites, and at the “critical AuNP mass” of a PIM, all these extraction sites are blocked and the membrane loses its ability to extract.

## 1. Introduction

Polymer inclusion membranes (PIMs) are a type of liquid membranes composed of a base-polymer and a membrane liquid phase consisting of an extractant (often referred to as the carrier) and in some cases a plasticizer or modifier [1,2,3]. The most frequently used base-polymers are poly(vinyl chloride) (PVC) and cellulose triacetate (CTA), but other base-polymers such as poly(vinylidene-fluoride-co-hexafluoropropylene) (PVDF-HFP) and semi-interpenetrating crosslinked PVDF-HFP poly(ethylene glycol) dimethacrylate (PVDF-HFP/PEG-DMA) have recently been found to provide a better performance—particularly in terms of their long-term stability [4,5,6,7,8]. The majority of the published research on PIMs has been focused on the extraction and transport of metallic cations and anions and small organic molecules (e.g., [9,10,11]). However, recent studies have demonstrated that PIMs are having an increasing role in chemical analysis techniques involving separation and sensing [12] and in the manufacturing of layers of metallic nanoparticles on membrane surfaces [13,14,15,16,17].

Successful PIMs are described as those that show good compatibility between the membrane components and the extracted complex or ion-pair of the target chemical species. Such membranes appear transparent and homogeneous to the naked eye, and are mechanically strong [18]. This evaluation of PIMs is useful in their practical application, but does not provide an insight into their morphology at micrometer- and nanometer-size scales. A commonly held view is that the liquid phase in a PIM is located between the entangled polymer chains in a network of nanometer-size channels, whereas the liquid phase in a supported liquid membrane (SLM)—the most popular type of liquid membranes at present—is held only by capillary forces within the micrometer-size pores of an inert polymeric membrane. It is suggested that this is the reason why PIMs are generally more stable than SLMs. Several advanced material characterization techniques (e.g., scanning electron microscopy (SEM), atomic force microscopy (AFM), X-ray diffraction (XRD), and Fourier transform infrared spectroscopy (FTIR)) were used in studies aimed at clarifying the morphology of PIMs [1,3]. Arous et al. [19] reported that the SEM images of a pure CTA-based membrane showed a highly porous matrix, but the pores vanished and a dense membrane was formed when 2-nitrophenol octyl ether (NPOE) was added as a plasticizer to the membrane composition. On the other hand, Xu et al. [20] reported that dense films were formed with no apparent porosity in PVC-based PIMs with a low concentration of Aliquat 336 as the carrier. As the concentration of Aliquat 336 was increased, the authors reported that a porous membrane structure with irregularly-shaped pores and pore sizes was observed. A more recent study by St John et al. [21] using synchrotron-based FTIR microspectrometry showed that PVC-based PIMs containing Aliquat 336 were chemically homogeneous at the micrometer-size scale.

The transport mechanism in PIMs is also still uncertain, and both the “mobile carrier” and the “fixed site” models have been proposed [1,2,3]. The “mobile carrier” model suggests that PIMs consist of liquid-filled nanometer-size channels, which are connected to extraction sites on the membrane surface, and that the extracted species diffuse through these surface extraction sites into the bulk of the PIM. However, no direct experimental evidence has yet been provided to support the existence of such extraction sites.

In a previous study, we reported on the use of a PVC-based PIM containing Aliquat 336 as a template for the preparation of surface-confined gold nanoparticles (AuNPs) [15]. The preparation process involved the extraction of Au(III) from an HCl solution and the subsequent reduction of the extracted Au(III) at the membrane surface with a solution of disodium ethylenediaminetetraacetate (Na_2_EDTA). When prepared under the appropriate conditions, the AuNPs provided maximum coverage of the PIM surface and the membrane became incapable of extracting chemical species. However, this phenomenon was not studied in detail in the previous work [15]. This raised the interesting question of whether the AuNPs were formed at extraction sites on the PIM surface and the PIM became extraction inactive due to the blocking of these sites. The present study was aimed at confirming the existence of such extraction sites and determining the “critical AuNP mass” at which all surface extraction sites were blocked by AuNPs.

## 2. Materials and Methods

### 2.1. Chemicals

All chemicals were used as received. Deionized water (18 MΩ cm, Millipore, Synergy 185, Molsheim, France) was used in the preparation of all aqueous solutions.

Aliquat 336 (Aldrich, a mixture of quaternary alkylammonium chlorides), high molecular weight powdered PVC (Fluka), 1-dodecanol (DD) (Aldrich), NPOE (Fluka), and tetrahydrofuran (THF) (Chem-supply) were used as received. Au(III) calibration standards were made from a 1000 mol·L^−1^ Au(III) stock solution (BDH Spectrosol).

Au(III) solutions for membrane extraction were prepared from HAuCl_4_ (Aldrich) dissolved in 2.5 M HCl (Chem-supply) solution. EDTA solutions of concentration 0.10 mol·L^−1^ were prepared from Na_2_EDTA (Fison), and the pH was adjusted to pH 6.0 with 1.0 M NaOH solution (Chem-supply) [15].

Nitrate solutions (100 mg·L^−1^) were prepared by dissolving KNO_3_ (Asia Pacific Specialty Chemicals) in deionized water.

### 2.2. Instrumentation

The concentration of Au(III) was determined by atomic absorption spectrometry (AAS) (Z-2000 Series Polarized Zeeman spectrometer, Hitachi, Tokyo, Japan). The extraction experiments were carried out by continuously shaking the nitrate solutions with a PIM immersed in each one of them. A platform orbital shaker (OM6, Ratek, Melbourne, Australia) was used in these experiments. The concentration of NO_3_^−^ was determined by ion chromatography (IC) (DX-120 ion chromatograph, Dionex, Sunnyvale, CA, USA) with the following experimental conditions: eluent—4.8 mM Na_2_CO_3_, 0.6 mM NaHCO_3_; flow rate—1.2 mL min^−1^; and sample loop size—25 µL.

A scanning electron microscope (Quanta 200 F, FEI, Zurich, Switzerland) was used for membrane imaging. Measurements were carried out at 20 kV in high vacuum. The resolution of this instrument as stated by the manufacturer is between 1.2 nm to 3.0 nm at 30 kV and 1 kV, respectively.

### 2.3. Membrane Preparation

A mixture of Aliquat 336, PVC, and in some cases a plasticizer (NPOE or DD) with a total mass of 400 mg was dissolved in a small volume of THF (8–10 mL), and the mixture was poured into a glass ring (diameter—7 cm) positioned on a flat glass plate. The mixture was covered with a filter paper and a watch glass to allow slow evaporation of the THF over 24 h to give a visually transparent, homogeneous, and flexible circular membrane. The membrane was then removed from the glass plate and cut with a metal cutter (diameter 3.5 mm). The cut edge was trimmed to give an average mass and thickness of 60 ± 3 mg and 50 ± 5 μm, respectively.

### 2.4. Preparation of AuNPs on the Surface of PIMs

The AuNP-coated PIMs were prepared by firstly immersing the membranes in individual flasks containing 100 mL of 100 mg·L^−1^ Au(III) (present as [AuCl_4_]^−^) and 2.5 M HCl and shaking them on a platform orbital shaker (150 rpm) for a predetermined period of time. Samples of the Au(III) solution (0.20 mL) were removed at the start and the end of the extraction period. The samples were diluted to 4 mL with deionized water, and the Au(III) concentration was determined by AAS. The Au(III)-loaded membranes were then rinsed with 5 mL of deionized water and dried before immersing them into 100 mL of 0.10 M EDTA solution at pH 6.0. The solutions were shaken on the platform orbital shaker for 24 h to reduce Au(III) to AuNPs on the surface of the PIMs.

### 2.5. Extraction of Nitrate

The AuNP-coated membranes and PIMs without AuNPs were immersed individually in 100 mL of 100 mol·L^−1^ NO_3_^−^ solutions in flasks which were shaken (150 rpm) on a platform orbital shaker. Samples of the NO_3_^−^ solution (1 mL) were removed at predetermined times throughout the course of the extraction experiment. The samples were diluted to 6 mL with deionized water, and the NO_3_^−^ concentration was determined by IC.

### 2.6. Recovery of Au from the AuNP-Coated PIMs

A centrifuge tube was dried in an oven at 35 °C overnight, and its mass was recorded. An AuNP-coated PIM was placed in it, and this was followed by the addition of 1.5 mL of THF. The tube was shaken and sonicated for 10 min in an ultrasonic bath until the membrane had dissolved, and the tube was centrifuged for 5 min at 8000 rpm to ensure that all AuNPs settled at the bottom of the tube. THF was then removed from the centrifuge tube with a pipette, and the precipitate was rinsed twice with 1 mL of THF to remove any traces of membrane material. The precipitate in the centrifuge tube was dried in an oven at 35 °C overnight, and the tube was reweighed to obtain the mass of metallic gold.

### 2.7. Initial Flux Calculation

The calculation of the initial flux (*J*_0_, mol·m^−2^·s^−1^) was made according to Fick’s first law by fitting the transient concentration with an exponential decay function ($\bar{C}=a_{1}+a_{2}e^{-a_{3}t}$), the first derivative of which (${\left(d\bar{C}/dt\right)}_{t=0}$) was used to calculate *J*_0_ according to Equation (1) [22].

(1) $$J_{0}=\frac{V}{S}\cdot {\left[\frac{dC}{dt}\right]}_{t=0}$$

where *V* is the solution volume (m^3^), *S* is the PIM surface area (m^2^), and *t* is time (s).

## 3. Results and Discussion

### 3.1. Formation of AuNPs on the PIM Surface

**Figure 1:**
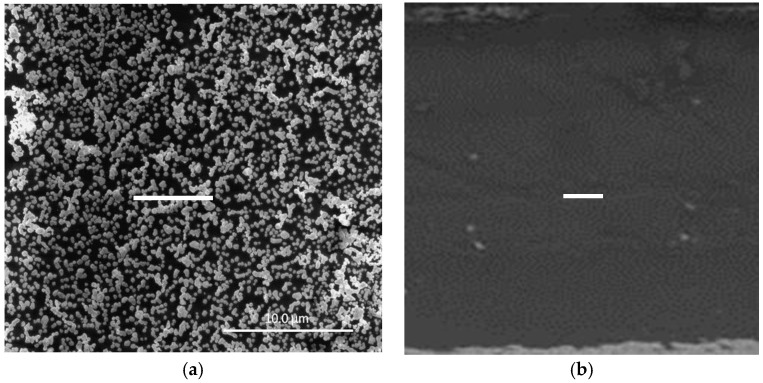
SEM images showing (**a**) surface-confined gold nanoparticle (AuNP) clusters at maximum Au(III) loading and (**b**) the cross-section of a polymer inclusion membrane (PIM) coated with AuNPs under the same conditions which is virtually free of AuNPs. The white bars are equal to 10 μm.

In a previous study [15], we demonstrated that surface-confined AuNPs on a PVC-based PIM could be produced by a straightforward procedure. In it, a PIM (20 wt % Aliquat 336, 10 wt % DD, and 70 wt % PVC) with an ion-exchange capacity of 0.40 meq g^−1^ was firstly immersed in a 2.5 M HCl solution of Au(III). Au(III) was extracted into the PIM as [AuCl_4_]^−^ and the amount extracted was determined by the experimental conditions; however, complete loading of the PIM with Au(III) can be easily obtained (Figure S1, Supplementary Material). The Au(III)-loaded PIM was then immersed in 1.0 M EDTA solution at pH 6 for 24 h, producing the surface-confined AuNPs. The SEM image (Figure 1a) of the surface of an AuNP-coated PIM with maximum Au(III) loading and prepared under the optimum conditions [15] shows that AuNPs were present on the surface of the PIM, where depending on their surface density they can aggregate to form clusters. The SEM image of the cross-section (Figure 1b) revealed the absence of AuNPs within the bulk of the membrane.

It was expected that the surface-confined AuNPs and clusters of those were likely to affect the extraction properties of the corresponding PIMs. This effect was experimentally studied using the nitrate ion as the extracted chemical species because Aliquat 336 had been shown to exhibit a relatively high affinity for this ion [23,24].

### 3.2. Extraction of NO_3_^−^ Using an AuNP-Coated PIM

**Figure 2:**
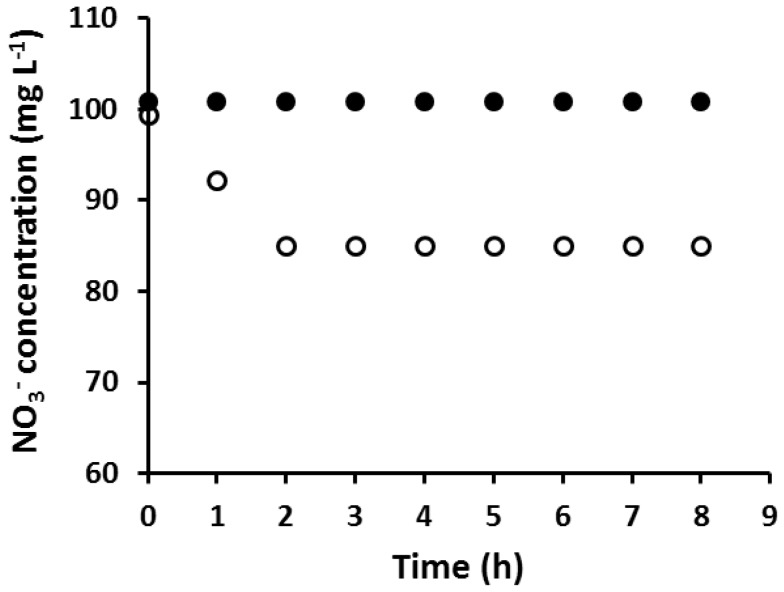
The extraction of NO_3_^–^ using an AuNP-coated PIM, fully loaded with Au(III) prior to reduction (●), and using a PIM of the same composition but without AuNPs (○). Experimental conditions: solution volume and composition: 100 mL, 100 mg·L^−1^ NO_3_^−^; PIM mass and composition: 60 ± 3 mg, 20 wt % Aliquat 336, 10 wt % 1-dodecanol (DD) and 70 wt % poly(vinyl chloride) (PVC); shaking rate: 150 rpm. Data points are the average of three extraction experiments with an average standard deviation (SD) of 0.77 mg·L^−1^.

An AuNP coated PIM which was fully loaded with Au(III) prior to its reduction with EDTA was found to be incapable of extracting NO_3_^−^, while a PIM with the same composition but without AuNPs extracted NO_3_^−^ as expected (Figure 2).

In order to explain this phenomenon, it is proposed that the PIM surface was not homogeneous and contained membrane liquid phase located at extraction sites, while the remainder of the PIM surface was free of the liquid phase. The inability of the PIM coated with AuNPs to extract NO_3_^−^ is due to blocking of these extraction sites by the AuNPs or their clusters, thus preventing the transfer of NO_3_^−^ ions to the bulk of the membrane. If this were the case, then the extent of coverage of the PIM surface by the AuNPs is crucial, and by lowering the extent of coverage, some of the extraction sites would be open and extraction of NO_3_^−^ would take place. This line of reasoning suggests that there should be a minimal surface coverage (which can be referred to as the “critical AuNP mass”), at which the membrane ceases to extract because of complete blockage of its surface extraction sites. Thus, a study was conducted to determine this “critical AuNP mass” and to investigate its dependence on the membrane composition.

**Figure 3:**
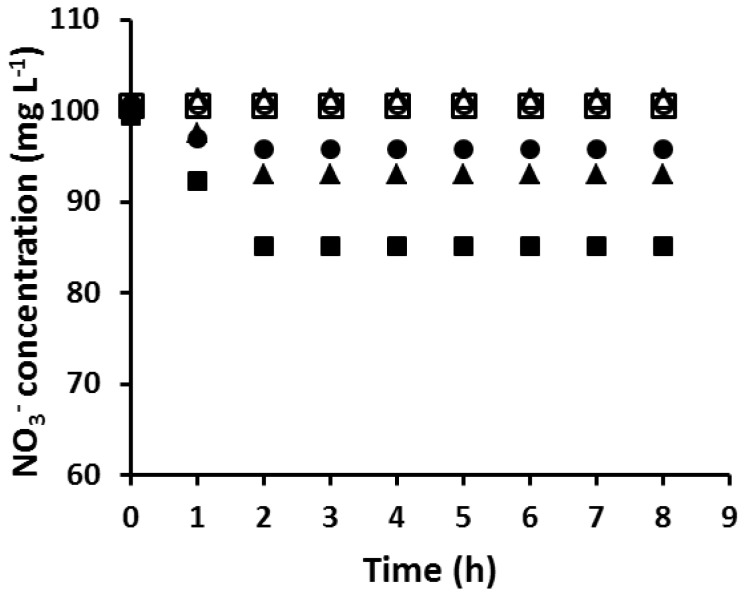
The extraction of NO_3_^−^ by the PIM with different AuNP masses and extraction times (∎, 0.00 mg/0 h; ▲, 3.57 mg/4 h; ●, 4.19 mg/8 h; □, 4.54 mg/12 h; △, 4.54/12.5 h; ○, 4.54 mg/79 h). Experimental conditions: solution volume and composition, 100 mL, 100 mol·L^−1^ NO_3_^−^; PIM mass and composition, 60 ± 3 mg, 20 wt % Aliquat 336, 10 wt % DD, and 70 wt % PVC; shaking rate: 150 rpm. Data points are the average of three extraction experiments with an average SD of 0.69 mg·L^−1^.

In order to determine the “critical AuNP mass” of a PIM composition, PIMs with the same composition were loaded with different amounts of Au(III) before reduction with EDTA. This was carried out by varying the time during which PIMs were immersed in the corresponding Au(III) solutions. The membranes were then tested for their ability to extract NO_3_^–^ and the results are shown in Figure 3. The mass of the AuNPs collected on the surface of the membrane was calculated by the method described in Section 2.6. The PIM which was not coated with AuNPs extracted an amount of NO_3_^−^ equivalent to its ion-exchange capacity of 0.40 meq g^−1^. However, as the amount of AuNPs on the membrane surface increased, the amount of NO_3_^−^ extracted into the PIMs decreased until the PIM became unable to extract nitrate. The critical mass of AuNPs to completely block the PIM surface was found to be 4.54 mg, which occurred at Au(III) extraction (loading) times equal to or greater than 12 h.

**Figure 4:**
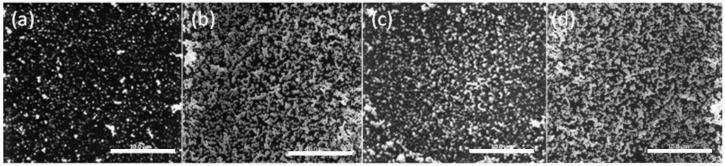
SEM images of the surface of the PIM corresponding to Au(III) extraction times of (**a**) 8 h; (**b**) 12 h; (**c**) 12.5 h; and (**d**) 79 h. The PIM loaded with Au(III) was exposed to 0.10 mol·L^−1^ ethylenediaminetetraacetate (EDTA) at pH 6.0 for 24 h. The white bars are equal to 10 μm.

The SEM images of the PIM surface for 8, 12, 12.5, and 79 h of extraction (loading) are shown in Figure 4.

The lower AuNP surface coverage of the PIM surface for 8 h (Figure 4a) is clearly evident, whereas for the other times the extent of coverage appears to be approximately the same and the mass of the AuNPs was the same (4.54 mg; i.e., “critical AuNP mass” for this PIM composition).

### 3.3. Quantitative Production of AuNPs on the PIM Surface

It was of interest to determine if the extracted Au(III) was quantitatively converted into AuNPs at the PIM surface as a result of the EDTA-based reduction process. This was carried out by using the procedure described in Section 2.6. The results were then compared with the amount of Au(III) extracted into the PIM during the Au(III) extraction process as determined by AAS. The possibility of the reduction of residual Au(III) in the membrane by THF itself was checked by dissolving Au(III)-loaded PIMs in THF. It was found that no metallic gold was produced in this way.

The average mass of Au(III) extracted into 10 identical PIMs (4.54 mg, SD of 0.15 mg, and 95% confidence interval of 4.43–4.64 mg) agreed very closely with the average mass of metallic Au collected by dissolving the same 10 PIMs in THF (4.52 mg, SD of 0.18 mg, and 95% confidence interval of 4.39–4.65 mg). There was no statistically significant difference between the two average masses at the 95% confidence level. These results confirmed that under the experimental conditions used, all extracted Au(III) was reduced to AuNPs on the membrane surface. This result indicated that all Aliquat 336 ion-pairs containing [AuCl_4_]^−^ as the anion in the bulk of the PIM prior to the reduction step were converted back to their original chloride form, and were therefore potentially available for extracting NO_3_^−^ [23,24].

### 3.4. Effect of the Aliquat 336 Concentration on the “Critical AuNP Mass”

PIMs with 20, 25, 30, and 35 wt % Aliquat 336 but without an additional plasticizer were studied to determine the effect of the Aliquat 336 concentration on the “critical AuNP mass”. As expected [15,25,26,27], the extent and rate of Au(III) extraction increased with increasing the concentration of Aliquat 336 in the PIM (Figure S2, Supplementary Material).

After extraction, each PIM was immersed in an EDTA solution to produce AuNPs and then tested for its ability to extract NO_3_^−^. The NO_3_^−^ extraction curves which also show the mass of metallic gold recovered from the membranes after its dissolution in THF are presented in the Supplementary Material (Figures S3–S6). The “critical AuNP mass” with the corresponding initial Au(III) flux value and “critical” Au(III) extraction (loading) time for each membrane composition are summarized in Table 1. In each case, extraction of NO_3_^−^ ceased once the “critical AuNP mass” was reached. Table 1 shows very clearly that an increase in the amount of Aliquat 336 in the PIM led to an increase in the “critical AuNP mass”.

**Figure 5:**
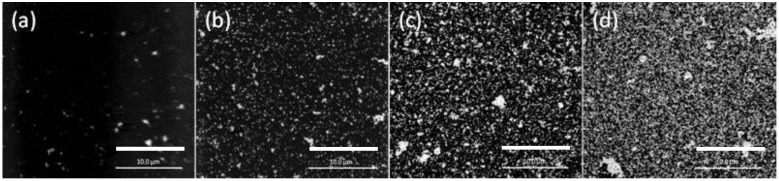
SEM images of the surfaces of the PIMs with a “critical AuNP mass” for the following concentrations of Aliquat 336: (**a**) 20; (**b**) 25; (**c**) 30; and (**d**) 35 wt %. The white bars are equal to 10 μm.

The SEM images of the surfaces of the PIMs listed in Table 1 are shown in Figure 5. The increase in the number of AuNP clusters with the increase in the concentration of Aliquat 336 is evident.

It can be expected that, if the “critical AuNPs mass” is related to the number of extraction sites on the PIM surface, then PIM compositions that produce a higher “critical AuNP mass” will exhibit faster extraction of Au(III); i.e., higher initial Au(III) flux values. This is certainly found to be the case for the four membrane compositions studied, as shown in Table 1.

### 3.5. Effect of DD and NPOE

In our previous studies [15,27], we optimized the PIM composition and found that the most efficient membrane for the extraction of Au(III) was obtained by adding DD to the PIM composition. The research described in Section 3.1, Section 3.2 and Section 3.3 involved the use of this PIM. Another common plasticizer used in PIM compositions is NPOE [1,2,3], and it was of interest to examine the effect of the concentrations of these two plasticizers on the “critical AuNP mass”. Plasticizers are generally employed to improve the compatibility of the membrane components and to improve the extraction rate and extraction efficiency [1,2,3]. In this study, the concentration of Aliquat 336 in the PIMs was kept constant at 20 wt % to be consistent with the previous studies [15,27], while the concentrations of DD or NPOE were varied to provide PIMs containing 0, 5, 10, and 15 wt % of plasticizer with the concentration of PVC being varied accordingly.

The PIMs were first loaded with Au(III) as described before. As reported previously [15], the extraction was faster for the PIMs containing DD, and these PIMs reached equilibrium with the solution after several hours. Additionally, there was little difference in the initial flux for 10 and 15 wt % DD. The extraction curves are presented in the Supplementary Material (Figures S7 and S8). After Au(III) loading, the PIMs were treated with an EDTA solution to produce surface-confined AuNPs.

The AuNP coated PIMs were then used in NO_3_^–^ extraction experiments (Figures S9–S13, Supplementary Material) to determine the “critical” Au(III) loading time and the corresponding “critical AuNP mass”. The data obtained are presented in Table 2. 

**Figure 6:**
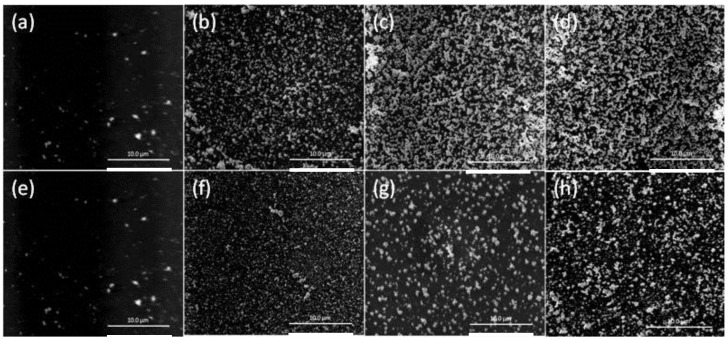
SEM surface images of the PIMs listed in Table 2. Aliquat 336, 20 wt %; DD, (**a**) 0; (**b**) 5; (**c**) 10; (**d**) 15 wt %; 2-nitrophenol octyl ether (NPOE); (**e**) 0; (**f**) 5; (**g**) 10; and (**h**) 15 wt %. The white bars are equal to 10 μm.

It can be seen that the “critical AuNP mass”, initial Au(III) flux, and “critical” Au(III) extraction time increased with increasing the concentrations of DD and NPOE, with DD producing a considerably higher “critical AuNP mass” value than NPOE. Additionally, the “critical AuNP masses” for both plasticizers was considerably higher than that for an un-plasticized PIM, thus suggesting the presence of more available surface extraction sites, which is consistent with the faster extraction of Au(III) by plasticized PIMs compared to un-plasticized PIMs [27]. Membranes containing concentrations of DD or NPOE higher than 15 wt % became mechanically weak and unstable, and often had an oily surface. The SEM surface images in Figure 6 of the PIMs listed in Table 2 clearly show these “critical AuNP mass” trends.

## 4. Conclusions

The research reported in this paper has produced a number of interesting observations which are consistent with the surface of PIMs studied incorporating an array of sites where extraction can only occur. 

The experimental observations made in this research are the following:

- Individual AuNPs that aggregate into clusters are formed on the surface of the PIMs after the extraction of Au(III) and its subsequent reduction with EDTA.
- At a critical surface mass of the AuNPs, the PIM loses its ability to extract NO_3_^−^, which is consistent with AuNPs and clusters of those completely blocking the extraction sites on the PIM surface. At AuNP masses lower than the corresponding critical values, some sites are still available for the extraction of NO_3_^−^, but in such cases, the rate of extraction is reduced accordingly.
- The mass of AuNPs collected from PIMs after dissolution in THF equates exactly to the mass of Au(III) originally extracted. This demonstrates that all Au(III) extracted has been reduced to AuNPs on the PIM surface and the bulk of the PIM contains free Aliquat 336.
- The “critical AuNP mass”, and hence the population of extraction sites, is directly related to the PIM composition. Higher concentrations of Aliquat 336 result in higher Au(III) fluxes during Au(III) extraction and higher “critical AuNP mass” values. Additionally, the addition of increasing concentrations of DD or NPOE to the PIM formulation produces higher Au(III) fluxes and “critical AuNP mass” values.

On the micrometer-size scale, PIMs appear to be homogeneous [21]. As mentioned in the introduction, a number of papers have suggested that a “pore” structure exists in PIMs; however, the resolution associated with most of the instrumental methods employed in studying PIM morphology is not high enough to define a “pore” structure in the nanometer-size range. It could be hypothesized that a PIM is characterized by a tortuous channel-like structure and that these channels end in “pores” at the surface of the membrane. However, without direct evidence, the hypothesis about the internal structure of PIMs is highly speculative, even though the research in this paper on the membrane surface morphology does lend some credence to it. 

The above conclusions and observations are also consistent with the following experimental evidence: 

(1) PIMs are generally more resistant to leaching of the liquid membrane phase to the aqueous phase they are in contact with. 

(2) PIMs have lower rates of extraction and transport than SLMs, since the pores in SLMs are generally in the micrometer-size range.

Research is continuing on this approach with a view to refining the preparation of the surface confined AuNPs and to obtain a higher resolution examination of both the PIM surface and its internal structure using SEM, AFM, XRD, and synchrotron-based techniques. The ultimate aim is to elucidate the true nature of the PIM surface and its internal structure.

## Figures and Tables

**Table 1:** “Critical AuNP masses”, initial Au(III) flux values, and “critical” Au(III) extraction (loading) times of PIMs for different concentrations of Aliquat 336.

Aliquat 336 Concentration (wt %)	Initial Flux for Au(III) Extraction (*J*_0_) (mol·m^−2^·s^−1^)	Critical Au(III) Extraction Time (h)	Critical AuNP Mass (mg)
20	3.12 × 10^−8^	2.5	0.16
25	1.56 × 10^−7^	3.5	0.45
30	5.20 × 10^−7^	6.5	3.45
35	2.60 × 10^−6^	7.5	5.44

**Table 2:** “Critical AuNP mass”, initial Au(III) flux, and “critical” Au(III) extraction (loading) time for PIMs containing 20 wt % Aliquat 336 and different concentrations of DD or NPOE.

DD/NPOE Concentration (wt %)	Initial Flux for Au(III) Extraction (J_0_) (mol·m^−2^·s^−1^)	Critical Au(III) Extraction Time (h)	Critical AuNP Mass (mg)
0	3.12 × 10^−8^	2.5	0.16
DD			
5	4.68 × 10^−7^	8	3.05
10	2.08 × 10^−6^	12	4.54
15	2.08 × 10^−6^	12	4.57
NPOE			
5	2.60 × 10^−7^	4.5	0.88
10	3.64 × 10^−7^	4.5	2.02
15	5.20 × 10^−7^	4.5	2.84

## Supplementary Materials

The following are available online at http://www.mdpi.com/2077-0375/8/1/6/s1: Figures S1–S13. Figure S1: The extraction of Au(III) from 2.5 mol·L^−1^ HCl. Experimental conditions: solution volume and composition, 100 mL, 100 mol·L^−1^ Au(III), 2.5 mol·L^−1^ HCl; PIM mass and composition, 60 ± 3 mg, 20% wt % Aliquat 336, 10 wt % 1-dodecanol, 70 wt % PVC; shaking rate, 150 rpm. Data points are the average of 3 extraction experiments with an average standard deviation (SD) of 0.77 mol·L^−1^. Figure S2: The extraction of Au(III) from 2.5 mol·L^−1^ HCl solutions into PIMs containing Aliquat 336 in concentrations: 20 (◆); 25 (■); 30 (▲); and 35 wt % (●) (Experimental conditions: solution volume and composition; 100 mL, 100 mol·L^−1^ Au(III), 2.5 mol·L^−1^ HCl; PIM mass: 60 ± 3 mg; shaking rate: 150 rpm). Data points are the average of 3 extraction experiments with an average SD of 0.79 mol·L^−1^. Figure S3: The extraction of NO_3_^−^ with PIMs of different AuNP loadings and extraction times. Experimental conditions: solution volume and composition; 100 mL, 100 mol·L^−1^ NO_3_^−^; PIM mass and composition: 60 ± 3 mg, 20 wt % Aliquat 336 and 80 wt % PVC; shaking rate: 150 rpm. Data points are the average of 3 extraction experiments with an average SD of 0.60 mol·L^−1^. Figure S4: The extraction of NO_3_^−^ with PIMs of different AuNP loadings and extraction times.Experimental conditions: solution volume and composition; 100 mL, 100 mol·L^−1^ NO_3_^−^; PIM mass and composition: 60 ± 3 mg, 25 wt % Aliquat 336 and 75 wt % PVC; shaking rate: 150 rpm. Data points are the average of 3 extraction experiments with an average SD of 0.62 mol·L^−1^. Figure S5: The extraction of NO_3_^−^with PIMs of different AuNP loadings and extraction times. Experimental conditions: solution volume and composition; 100 mL, 100 mol·L^−1^ NO_3_^−^; PIM mass and composition: 60 ± 3 mg, 30 wt % Aliquat 336 and 70 wt % PVC; shaking rate: 150 rpm. Data points are the average of 3 extraction experiments with an average SD of 0.62 mol·L^−1^. Figure S6: The extraction of NO_3_^−^ with PIMs of different AuNP loadings and extraction times. Experimental conditions: solution volume and composition; 100 mL, 100 mol·L^−1^ NO_3_^−^; PIM mass and composition: 60 ± 3 mg, 35 wt % Aliquat 336 and 65 wt % PVC; shaking rate: 150 rpm). Data points are the average of 3 extraction experiments with an average SD of 0.62 mol·L^−1^. Figure S7: The extraction of Au(III) from 2.5 mol·L^−1^ HCl solutions into PIMs containing 20 wt % Aliquat 336 and 0 (■), 5 (◆), 10 (●), and 15 wt % (▲) 1-dodecanol. Experimental conditions: solution volume and composition; 100 mL, 100 mol·L^−1^ Au(III), 2.5 mol·L^−1^ HCl; PIM mass: 60 ± 3 mg; shaking rate: 150 rpm. Data points are the average of 3 extraction experiments with an average SD of 0.73 mol·L^−1^. Figure S8: The extraction of Au(III) from 2.5 mol·L^−1^ HCl solutions into PIMs containing 20 wt % Aliquat 336 and 0 (■), 5 (◆), 10 (●), and 15 wt % (▲) NPOE. Experimental conditions: solution volume and composition; 100 mL, 100 mol·L^−1^ Au(III), 2.5 mol·L^−1^ HCl; PIM mass: 60 ± 3 mg; shaking rate: 150 rpm. Data points are the average of 3 extraction experiments with an average SD of 0.70 mol·L^−1^. Figure S9: The extraction of NO_3_^−^ with PIMs of different AuNP loadings and extraction times. Experimental conditions: solution volume and composition; 100 mL, 100 mol·L^−1^ NO_3_^−^; PIM mass and composition: 60 ± 3 mg, 20 wt % Aliquat 336, 5 wt % 1-dodecanol and 75 wt % PVC; shaking rate: 150 rpm. Data points are the average of 3 extraction experiments with an average SD of 0.69 mol·L^−1^. Figure S10: The extraction of NO_3_^−^ with PIMs of different AuNP loadings and extraction times. Experimental conditions: solution volume and composition; 100 mL, 100 mol·L^−1^ NO_3_^−^; PIM mass and composition: 60 ± 3 mg, 20 wt % Aliquat 336, 15 wt % 1-dodecanol and 55 wt % PVC; shaking rate: 150 rpm. Data points are the average of 3 extraction experiments with an average SD of 0.67 mol·L^−1^. Figure S11: The extraction of NO_3_^−^ with PIMs of different AuNP loadings and extraction times. Experimental conditions: solution volume and composition; 100 mL, 100 mol·L^−1^ NO_3_^−^; PIM mass and composition: 60 ± 3 mg, 20 wt % Aliquat 336, 5 wt % NPOE and 75 wt % PVC; shaking rate: 150 rpm. Data points are the average of 3 extraction experiments with an average SD of 0.68 mol·L^−1^. Figure S12: The extraction of NO_3_^−^ with PIMs of different AuNP loadings and extraction times. Experimental conditions: solution volume and composition; 100 mL, 100 mol·L^−1^ NO_3_^−^; PIM mass and composition: 60 ± 3 mg, 20 wt % Aliquat 336, 10 wt % NPOE and 70 wt % PVC; shaking rate: 150 rpm. Data points are the average of 3 extraction experiments with an average SD of 0.65 mol·L^−1^. Figure S13: The extraction of NO_3_^−^ with PIMs of different AuNP loadings and extraction times. Experimental conditions: solution volume and composition; 100 mL, 100 mol·L^−1^ NO_3_^−^; PIM mass and composition: 60 ± 3 mg, 20 wt % Aliquat 336, 15 wt % NPOE and 65 wt % PVC; shaking rate: 150 rpm. Data points are the average of 3 extraction experiments with an average SD of 0.67 mol·L^−1^.
