# Supplementary material for: The Effect of Surface Confined Gold Nanoparticles in Blocking the Extraction of Nitrate by PVC-Based Polymer Inclusion Membranes Containing Aliquat 336 as the Carrier
Source: Membranes (Basel). 2018 Jan 25;8(1):6. doi: 10.3390/membranes8010006 (PMC5872188; doi:10.3390/membranes8010006)
Supplement: Supplementary file 1 [file membranes-08-00006-s001.pdf]

Supplementary Material

The Effect of Surface Confined Gold Nanoparticles in Blocking the Extraction of Nitrate by PVC-Based Polymer Inclusion Membranes Containing Aliquat 336 as the Carrier

Ya Ya N. Bonggotgetsakul, Robert W. Cattrall and Spas D. Kolev *

School of Chemistry, The University of Melbourne, Victoria 3010, Australia;
E-Mails: yayabong88@gmail.com (Y.Y.N.B.); r.cattrall@unimelb.edu.au (R.W.C.).
* Correspondence: s.kolev@unimelb.edu.au; Tel.: +61-383-447-931

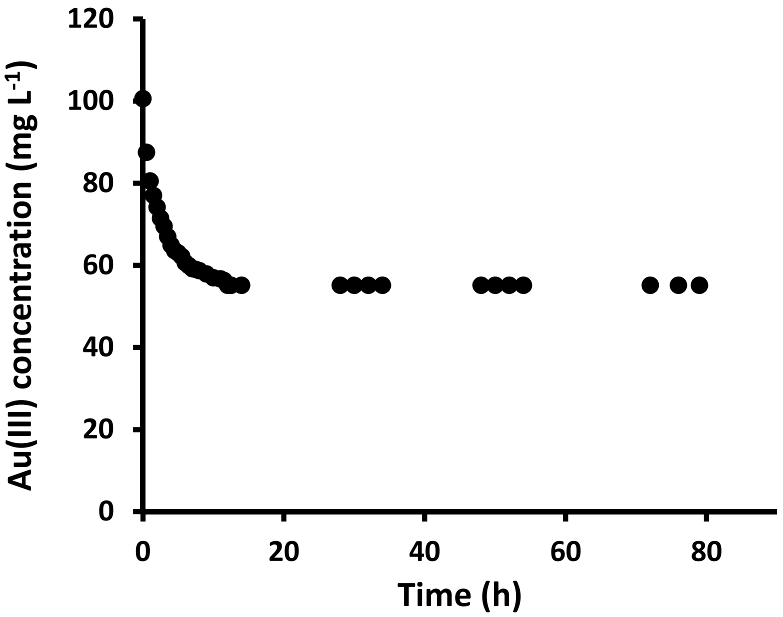

Figure S1. The extraction of Au(III) from 2.5 mol L^{-1} HCl. Experimental conditions: solution volume and composition, 100 mL, 100 mg L^{-1} Au(III), 2.5 mol L^{-1} HCl; PIM mass and composition, 60 ± 3 mg, 20% wt% Aliquat 336, 10 wt% 1-dodecanol, 70 wt% PVC; shaking rate, 150 rpm. Data points are the average of 3 extraction experiments with an average standard deviation (SD) of 0.77 mg L^{-1}.

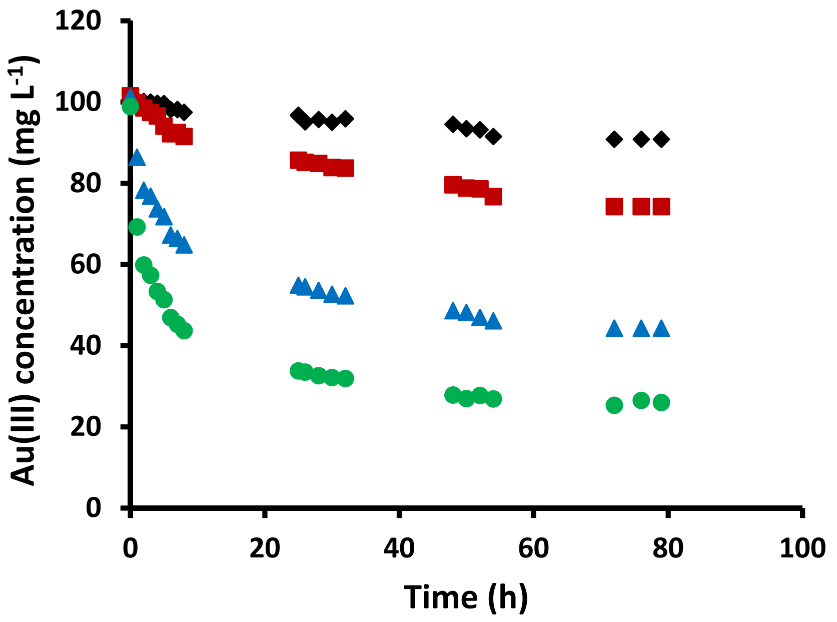

Figure S2. The extraction of Au(III) from 2.5 mol L^{-1} HCl solutions into PIMs containing Aliquat 336 in concentrations: 20 (◆); 25 (■); 30 (▲); and 35 wt% (●) (Experimental conditions: solution volume and composition; 100 mL, 100 mg L^{-1} Au(III), 2.5 mol L^{-1} HCl; PIM mass: 60 ± 3 mg; shaking rate: 150 rpm). Data points are the average of 3 extraction experiments with an average SD of 0.79 mg L^{-1}.

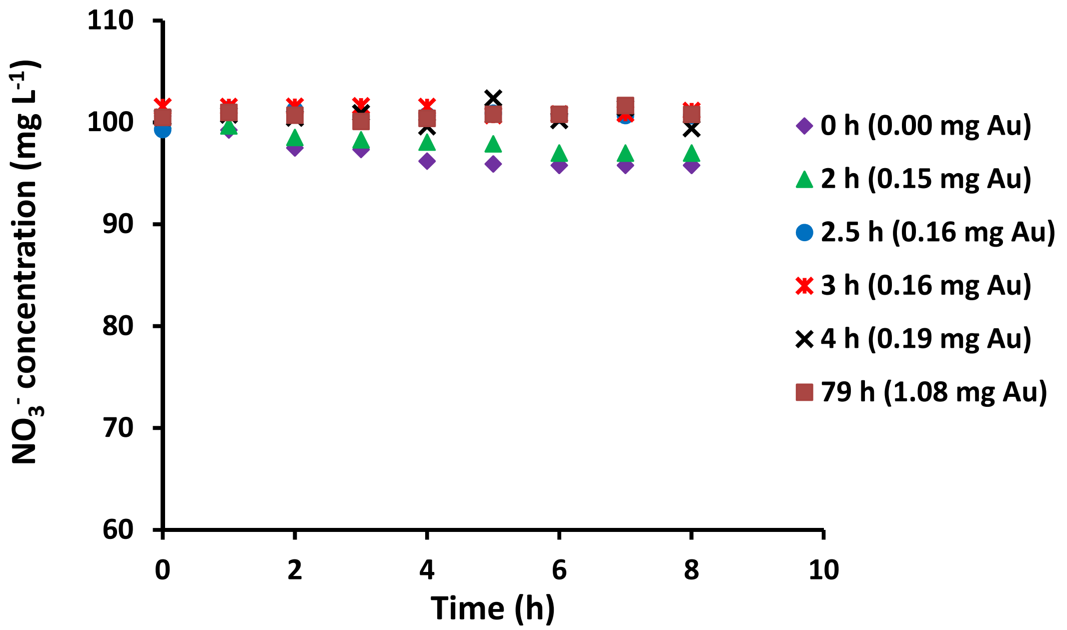

Figure S3. The extraction of NO_3^- with PIMs of different AuNP loadings and extraction times. Experimental conditions: solution volume and composition; 100 mL, 100 mg L^{-1} NO_3^-; PIM mass and composition: 60 ± 3 mg, 20 wt% Aliquat 336 and 80 wt% PVC; shaking rate: 150 rpm. Data points are the average of 3 extraction experiments with an average SD of 0.60 mg L^{-1}.

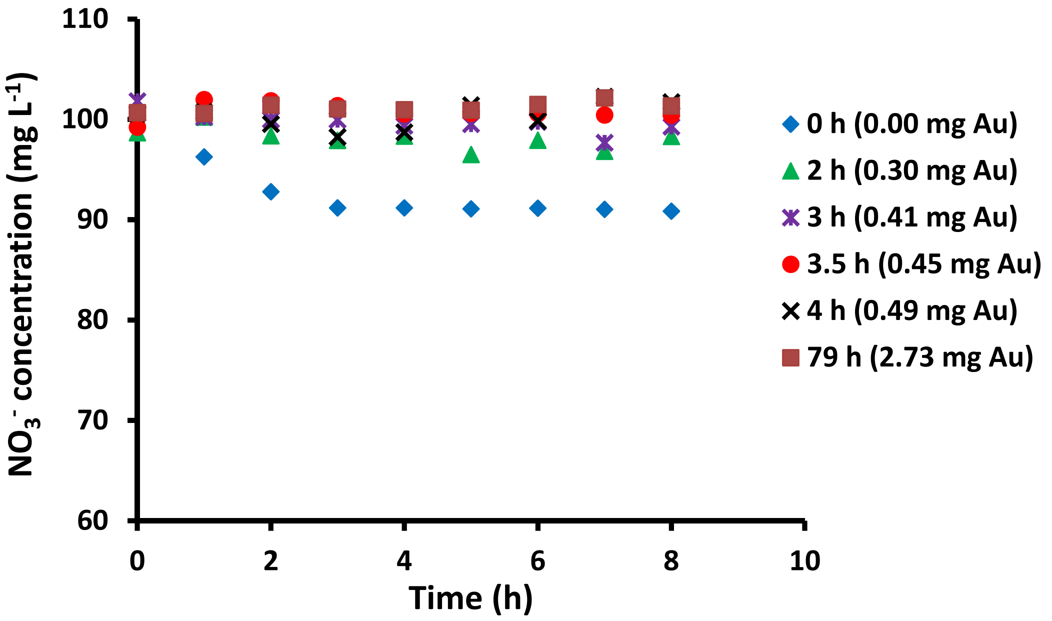

Figure S4. The extraction of NO_3^- with PIMs of different AuNP loadings and extraction times. Experimental conditions: solution volume and composition; 100 mL, 100 mg L^{-1} NO_3^-; PIM mass and composition: 60 ± 3 mg, 25 wt% Aliquat 336 and 75 wt% PVC; shaking rate: 150 rpm. Data points are the average of 3 extraction experiments with an average SD of 0.62 mg L^{-1}.

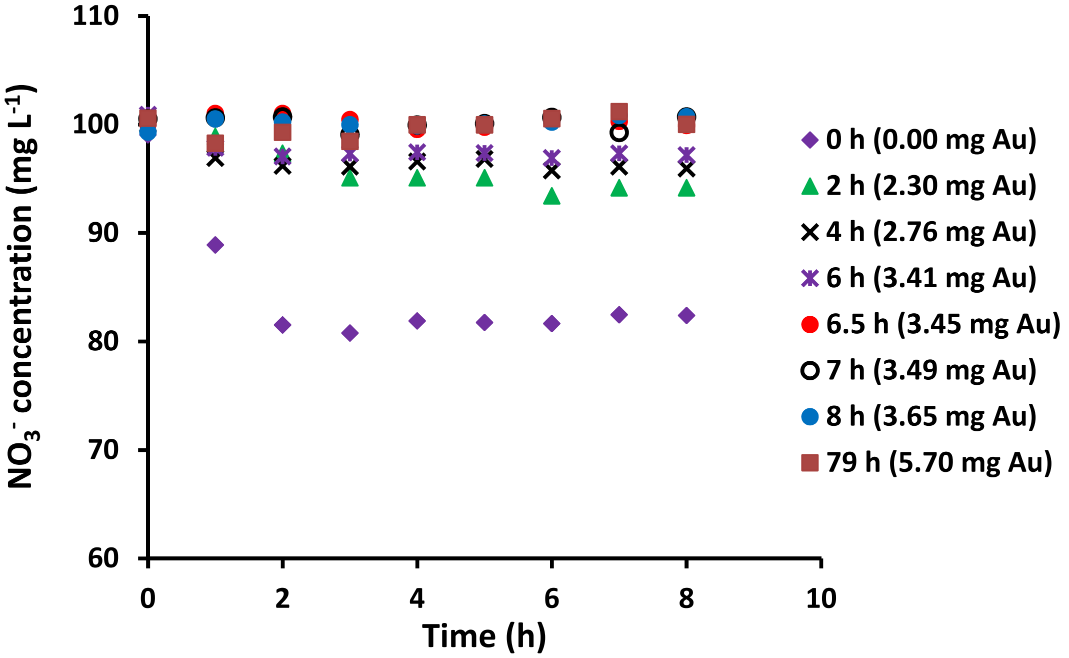

Figure S5. The extraction of NO_3^- with PIMs of different AuNP loadings and extraction times. Experimental conditions: solution volume and composition; 100 mL, 100 mg L^{-1} NO_3^-; PIM mass and composition: 60 ± 3 mg, 30 wt% Aliquat 336 and 70 wt% PVC; shaking rate: 150 rpm. Data points are the average of 3 extraction experiments with an average SD of 0.62 mg L^{-1}.

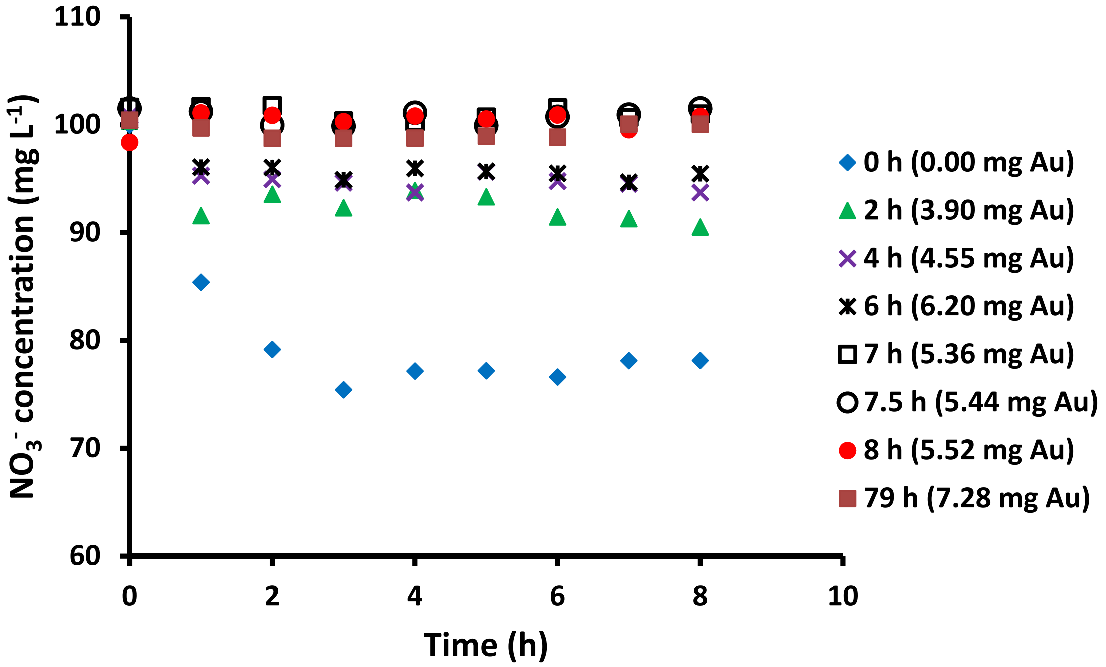

Figure S6. The extraction of NO_3^- with PIMs of different AuNP loadings and extraction times. Experimental conditions: solution volume and composition; 100 mL, 100 mg L^{-1} NO_3^-; PIM mass and composition: 60 ± 3 mg, 35 wt% Aliquat 336 and 65 wt% PVC; shaking rate: 150 rpm). Data points are the average of 3 extraction experiments with an average SD of 0.62 mg L^{-1}.

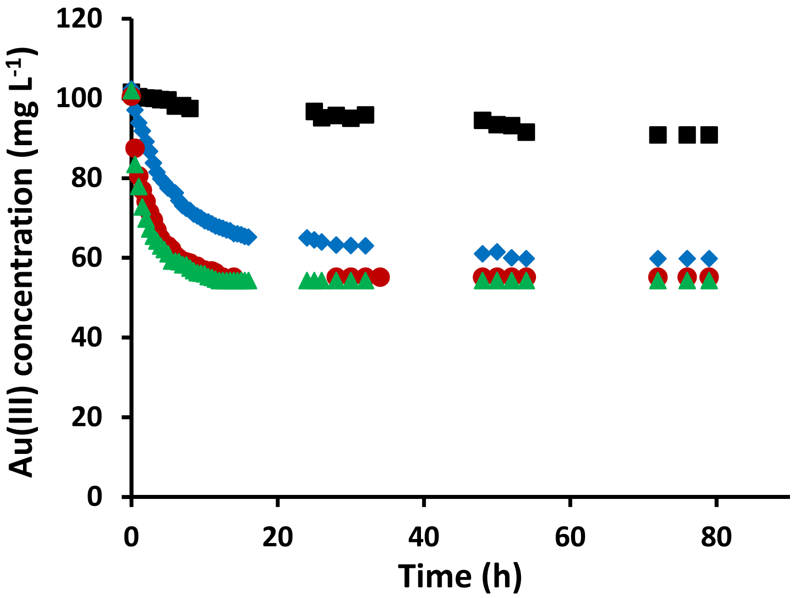

Figure S7. The extraction of Au(III) from 2.5 mol L^{-1} HCl solutions into PIMs containing 20 wt% Aliquat 336 and 0 (■), 5 (◆), 10 (●), and 15 wt% (▲) 1-dodecanol. Experimental conditions: solution volume and composition; 100 mL, 100 mg L^{-1} Au(III), 2.5 mol L^{-1} HCl; PIM mass: 60 ± 3 mg; shaking rate: 150 rpm. Data points are the average of 3 extraction experiments with an average SD of 0.73 mg L^{-1}.

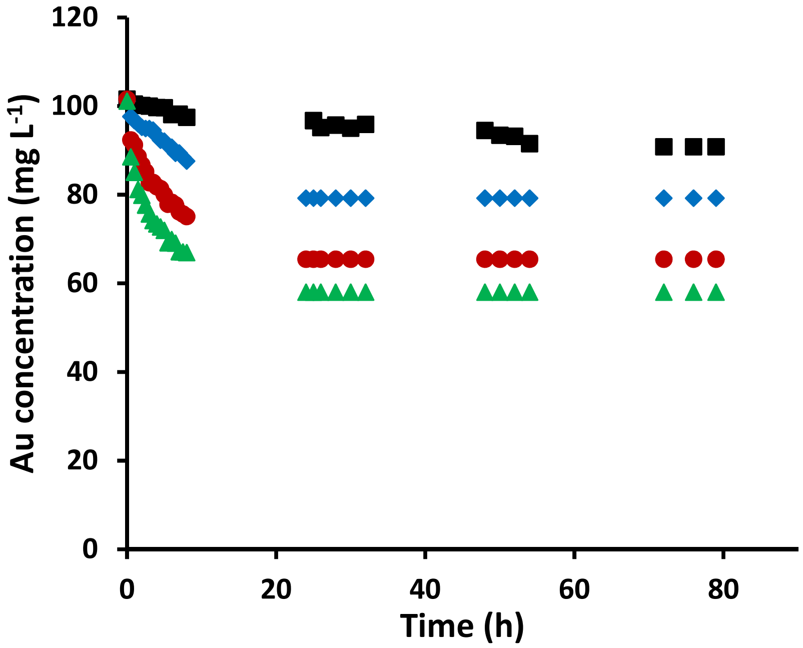

Figure S8. The extraction of Au(III) from 2.5 mol L^{-1} HCl solutions into PIMs containing 20 wt% Aliquat 336 and 0 (■), 5 (◆), 10 (●), and 15 wt% (▲) NPOE. Experimental conditions: solution volume and composition; 100 mL, 100 mg L^{-1} Au(III), 2.5 mol L^{-1} HCl; PIM mass: 60 ± 3 mg; shaking rate: 150 rpm. Data points are the average of 3 extraction experiments with an average SD of 0.70 mg L^{-1}.

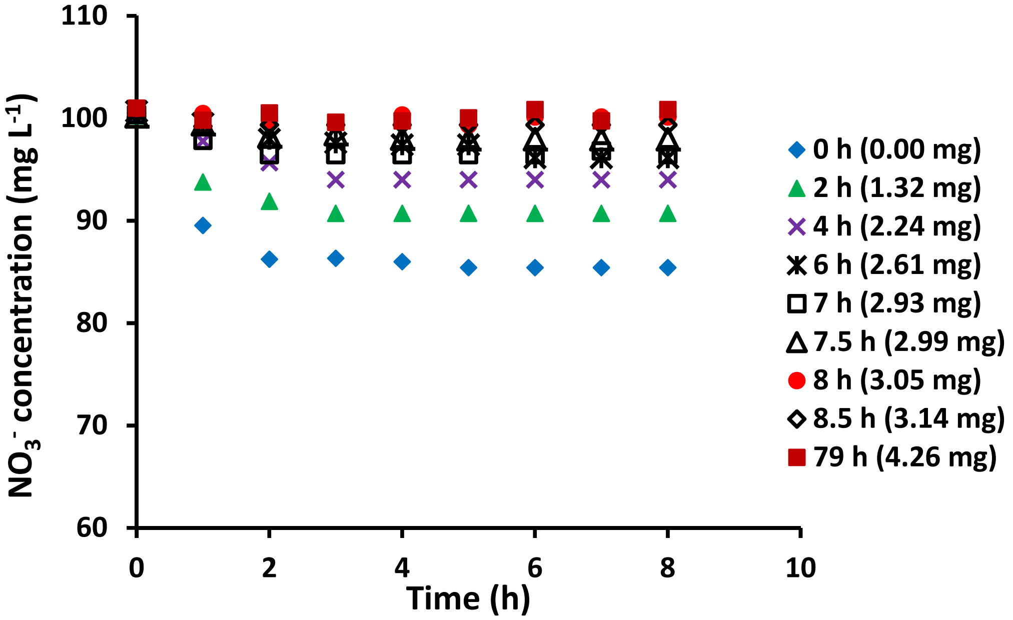

Figure S9. The extraction of NO_3^- with PIMs of different AuNP loadings and extraction times. Experimental conditions: solution volume and composition; 100 mL, 100 mg L^{-1} NO_3^-; PIM mass and composition: 60 ± 3 mg, 20 wt% Aliquat 336, 5 wt% 1-dodecanol and 75 wt% PVC; shaking rate: 150 rpm. Data points are the average of 3 extraction experiments with an average SD of 0.69 mg L^{-1}.

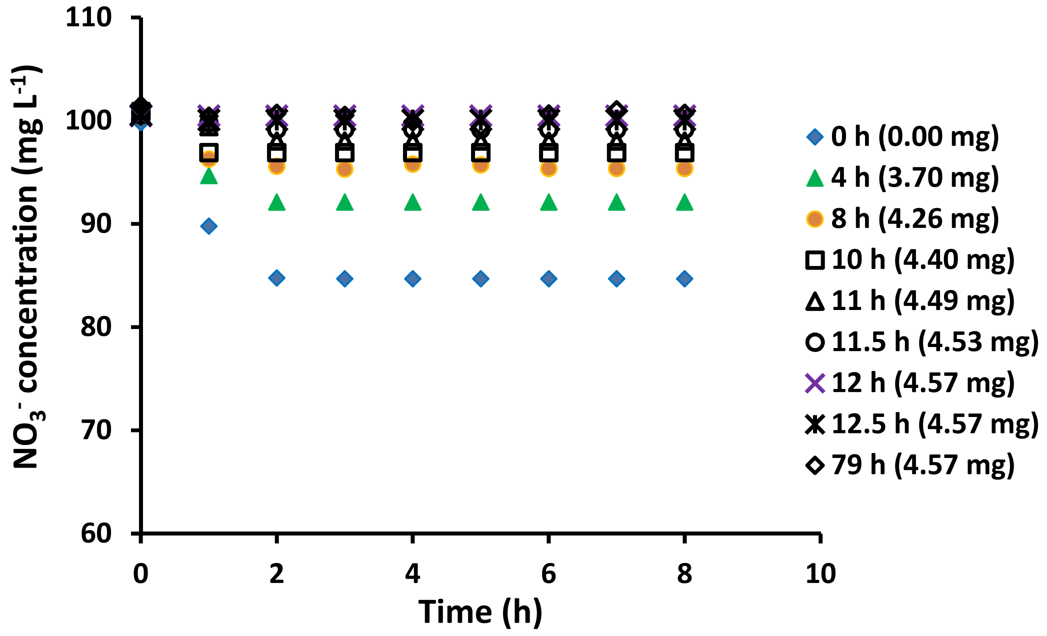

Figure S10. The extraction of NO_3^- with PIMs of different AuNP loadings and extraction times. Experimental conditions: solution volume and composition; 100 mL, 100 mg L^{-1} NO_3^-; PIM mass and composition: 60 ± 3 mg, 20 wt% Aliquat 336, 15 wt% 1-dodecanol and 55 wt% PVC; shaking rate: 150 rpm. Data points are the average of 3 extraction experiments with an average SD of 0.67 mg L^{-1}.

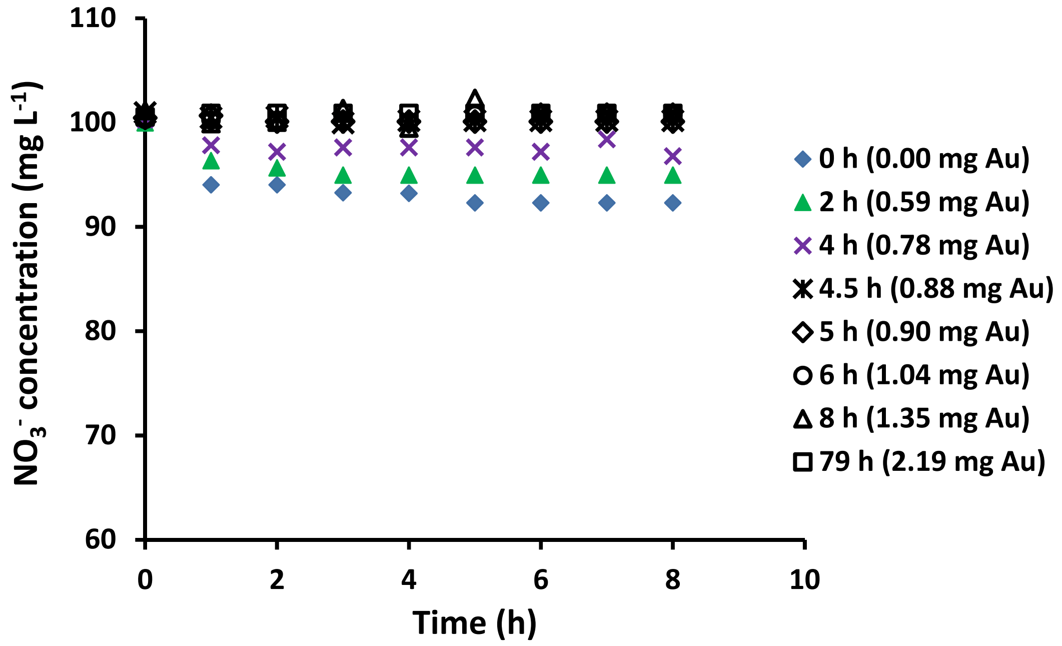

Figure S11. The extraction of NO_3^- with PIMs of different AuNP loadings and extraction times. Experimental conditions: solution volume and composition; 100 mL, 100 mg L^{-1} NO_3^-; PIM mass and composition: 60 ± 3 mg, 20 wt% Aliquat 336, 5 wt% NPOE and 75 wt% PVC; shaking rate: 150 rpm. Data points are the average of 3 extraction experiments with an average SD of 0.68 mg L^{-1}.

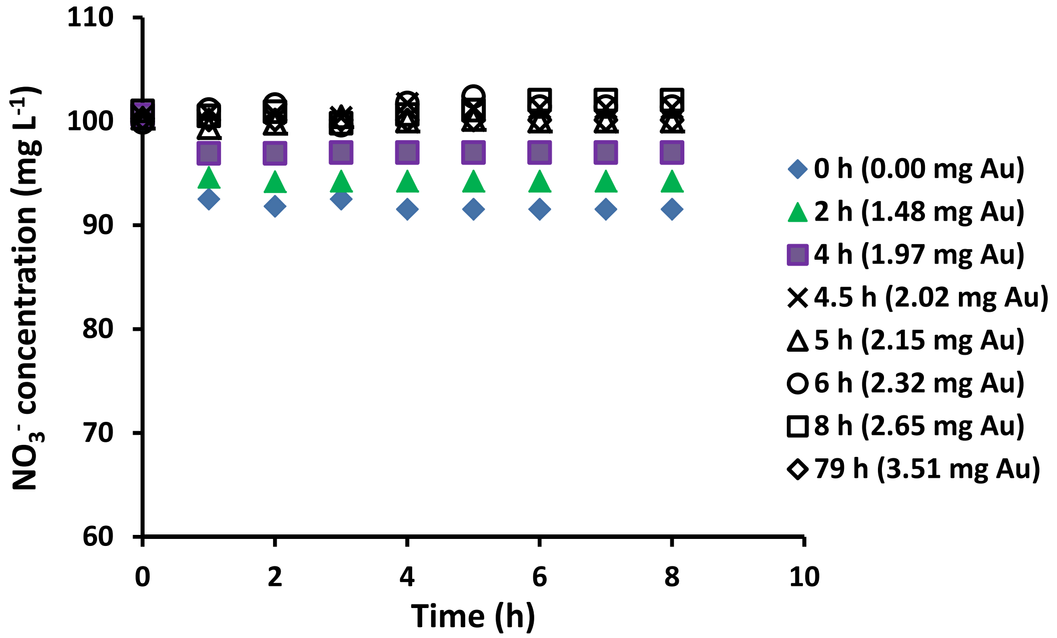

Figure S12. The extraction of NO_3^- with PIMs of different AuNP loadings and extraction times. Experimental conditions: solution volume and composition; 100 mL, 100 mg L^{-1} NO_3^-; PIM mass and composition: 60 ± 3 mg, 20 wt% Aliquat 336, 10 wt% NPOE and 70 wt% PVC; shaking rate: 150 rpm. Data points are the average of 3 extraction experiments with an average SD of 0.65 mg L^{-1}.

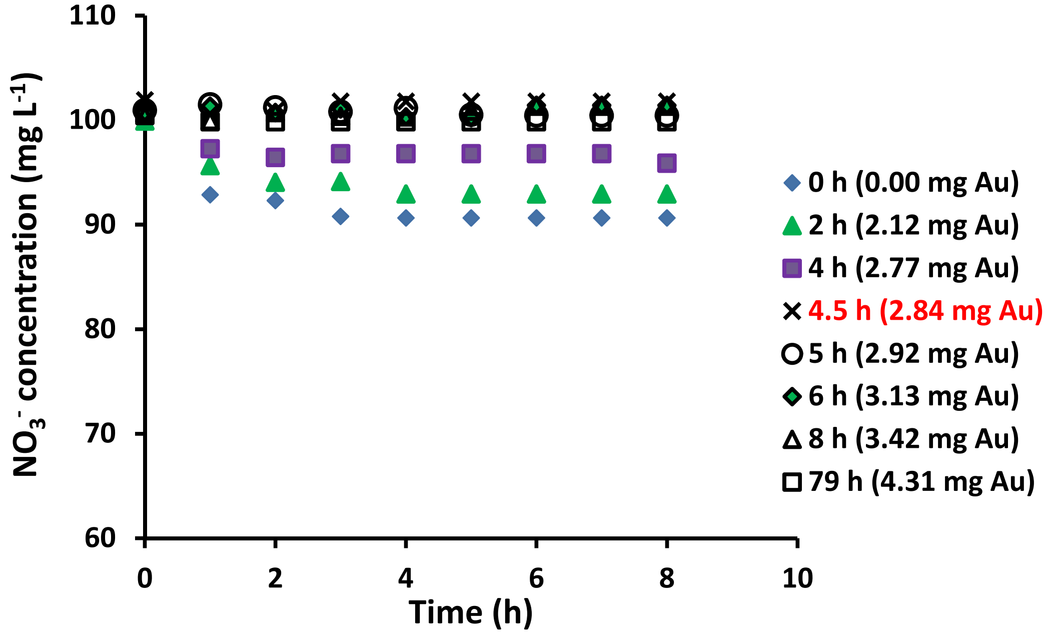

Figure S13. The extraction of NO_3^- with PIMs of different AuNP loadings and extraction times. Experimental conditions: solution volume and composition; 100 mL, 100 mg L^{-1} NO_3^-; PIM mass and composition: 60 ± 3 mg, 20 wt% Aliquat 336, 15 wt% NPOE and 65 wt% PVC; shaking rate: 150 rpm. Data points are the average of 3 extraction experiments with an average SD of 0.67 mg L^{-1}.
